# Peroxisome proliferator activated receptor γ protein expression is asymmetrically distributed in primary lung tumor and metastatic to lung osteosarcoma samples and does not correlate with gene methylation

**DOI:** 10.1186/s12917-015-0547-x

**Published:** 2015-09-04

**Authors:** Chamisa L. Herrera, Dae Young Kim, Senthil R. Kumar, Jeffrey N. Bryan

**Affiliations:** Veterinary Medicine and Surgery, College of Veterinary Medicine, University of Missouri, Columbia, MO 65211 USA; Veterinary Medical Diagnostic Laboratory, College of Veterinary Medicine, University of Missouri, Columbia, MO 65211 USA; Current Address: BluePearl Seattle, 11536 Lake City Way NE, Seattle, WA 98125 USA

**Keywords:** Peroxisome proliferator activated receptor-γ (PPAR-γ), Epigenetics, DNA Methylation, Lung cancer, Rosiglitazone

## Abstract

**Background:**

Peroxisome proliferator activated receptor-γ (PPAR-γ) is a ligand-dependent transcription factor that plays important roles in cellular proliferation and differentiation. It has been implicated as a tumor suppressor in many solid tumors including human prostate, breast, colon, and lung cancer. The objective of this study was to determine the tissue distribution of PPAR-γ in normal canine lung, canine lung cancer, and metastatic to lung cancer, as well as determine the role, if any, of DNA methylation in epigenetic control of gene expression. The protein was studied using immunohistochemistry (IHC) and DNA methylation was studied using combined bisulfite restriction analysis (COBRA), and methylation-specific PCR (MSP).

**Results:**

PPAR-γ is expressed in all large conducting airways, particularly in goblet cells and bronchial glands, in the canine lung. The protein is also expressed in interstitial macrophages. PPAR-γ is expressed in 33 % of canine non-small cell lung cancer (NSCLC) cases and 66 % of metastatic osteosarcoma (OSA) cases. There is a significant loss of 5′ PPAR-γ methylation from normal lung to primary lung cancer and metastatic OSA (*p* = 0.0002), however altered *PPAR-γ* promoter methylation at the interrogated locus does not appear to be associated with changes in protein expression.

**Conclusions:**

PPAR-γ protein is expressed in normal canine lung tissue, canine primary lung cancer, and metastatic OSA. Confirmation of PPAR-γ protein expression in tumor-bearing dogs supports the investigation of PPAR-γ agonists in this subset of veterinary patients. These results are the first to describe epigenetic marks and protein localization of PPAR-γ among different lung pathologies in the dog.

## Background

Peroxisome proliferator activated receptor-γ (PPAR-γ) is one of three members (α, β, γ) of the PPAR nuclear hormone receptor superfamily of ligand-dependent transcription factors. Natural ligands for PPAR-γ include fatty acids and eicosanoids [1]. PPAR-γ expression is prominent in adipocytes and its function is best described in regulating lipid metabolism, but PPAR-γ plays a more general role in cellular proliferation, differentiation, and survival, as well as acting as a negative regulator of inflammation [2, 3]. More recently, it has also been implicated as a tumor suppressor gene [4], and appears dysregulated in many human cancers including those of prostate, breast, colon, and lung. Research is rapidly discovering carcinogenic processes in which PPAR-γ is altered at the epigenetic, genetic and protein levels [5].

One of the most active areas of research is examining the role of PPAR-γ in lung cancer. Both human and murine studies have demonstrated that up-regulation of PPAR-γ can slow lung tumor development via reduced proliferation, decreased production of inflammatory cytokines, and promotion of a more differentiated phenotype [6–8]. Currently, lung cancer is the most common human cancer in the world and the leading cause of cancer related death [9]. Primary lung cancer (PLC) is divided into small-cell lung cancer (SCLC) and non-small cell lung cancer (NSCLC) with NSCLC predominating. Despite many advances in human oncologic surgery and chemotherapy, the 5 year survival rate for NSCLC remains lower than 15 % [9]. Due to these dismal survival rates, new approaches to cancer therapy are being investigated, including modulation of PPAR-γ. In fact, several studies have shown that artificial activation of PPAR-γ can inhibit growth of lung cancer cells, primarily through differentiation and apoptosis [10, 11].

The dog has proven repeatedly to be an excellent translational model for many human cancers. The dog shares many similarities with humans in respect to genetic aberrations leading to cancer, development of naturally occurring histologically similar cancers, environmental exposures, and similar responses to treatments including radiation, chemotherapy, and monoclonal antibody immunotherapy [12, 13]. Although rare in the dog, the species does serve as a good translational model for NSCLC in that dogs naturally develop NSCLC, are exposed to similar environmental inhalants, have a similar respiratory system anatomy, and similar size and distribution of primary lung tumors [14]. In addition, some genetic aberrations that occur in human NSCLC have also been documented in the dog including k-ras mutations and altered expression of proteins associated with chemotherapy resistance [15, 16]. And finally, the dog has also been used to demonstrate efficacy of new lung cancer therapies in humans, including use of inhalant chemotherapy [17, 18]. There is limited research describing PPAR-γ expression in the dog in health or disease. Information is specifically lacking in the contribution of this gene to carcinogenesis. Alterations in PPAR-γ expression have, however, been implicated in canine testicular tumors and nasal carcinomas [19, 20].

There are no reports of PPAR-γ expression in the canine lung or canine lung cancer. PPAR-γ agonists in the thiazolidinedione class (predominantly rosiglitazone) have been studied preliminarily in the dog, including pharmacokinetics and metabolism of this drug [21, 22]. More intriguing is that recent research suggests that the combination of PPAR-γ agonists with platinum based chemotherapy are synergistic in treating NSCLC *in vitro* for human NSCLC and *in vivo* in murine models. Additionally, safety of oral rosiglitazone and carboplatin was recently published in a phase I clinical trial for cancer-bearing dogs [23]. Given this information, dogs may serve as an excellent model of naturally occurring NSCLC to study the efficacy and tolerability of combination PPARγ agonists and platinum-based drugs for treatment of lung cancer. The protein may also be important in metastatic cancers like osteosarcoma, but this has not been evaluated in the dog.

Given that dogs develop NSCLC and are a good *in vivo* model for studying the effects of PPAR-γ agonists, it is critical to understand the expression of this protein and possible epigenetic control in the normal canine lung and canine lung cancer. The objective of this investigation was to identify PPAR-γ expression at the protein and epigenetic level in NSCLC and normal lung. In addition, as some protein alterations are necessary for carcinogenesis, and some are cancer specific, we also evaluated metastatic to lung osteosarcoma, to serve as an aggressive model of metastatic to lung cancer and a cancer control. The protein was studied in these groups through immunohistochemistry (IHC) and promoter methylation was studied using combined bisulfite restriction analysis (COBRA), and methylation-specific PCR (MSP). The hypotheses were that PPAR-γ would act as a tumor suppressor, and therefore have decreased expression with increasing malignancy, and that this would correspond with more complete 5′ PPAR-γ methylation.

## Results

### Patient demographics

The median ages of dogs were as follows: 8.3 years for the control group, 11.8 years for the NSCLC group, and 8.4 years for the OSA group. The NSCLC group was statistically older than both the OSA and control groups (p: 0.002 and 0.001 respectively). There was no statistical difference in gender between any of the groups (*p* = 0.658) (Table 1).

**Table 1:** Signalment and immunohistochemistry summary for all subjects evaluated

Dog	Age (years)	Sex	Breed	Conducting Airway^b^	Macrophages^b^	Tumor^b^	H&E Histologic Diagnosis
C-1	14	MC	Border Collie	n/a	(−)	n/a	Normal Lung
C-2	4	MC	Australian Shepherd	n/a	(−)	n/a	Normal Lung
C-3	7	MC	Mixed	n/a	(−)	n/a	Normal Lung
C-4	5	FS	Mixed	(+)	(−)	n/a	Normal Lung
C-5	8	MC	Dachshund	(+)	(−)	n/a	Normal Lung
C-6	8	MC	German Shepherd	n/a	(−)	n/a	Normal Lung
C-7	5	FS	Dachshund	n/a	(−)	n/a	Normal Lung
C-9	7	FS	Mixed	n/a	(−)	n/a	Normal Lung
C-10	7	MC	Doberman Pinscher	n/a	(−)	n/a	Normal Lung
C-15	13	MI	Mixed	(+)	(−)	n/a	Normal Lung
C-17	9	FS	Labrador Retriever	(+)	(−)	n/a	Normal Lung
C-18	7	FS	Shih Tzu	n/a	(−)	n/a	Normal Lung
C-19	9	MC	Boxer	n/a	(−)	n/a	Normal Lung
C-20	10	FI	Labrador Retriever	n/a	(−)	n/a	Normal Lung
C-21	12	MC	West Highland Terrier	(+)	(−)	n/a	Normal Lung
C-22	9	MC	Mixed	n/a	(−)	n/a	Normal Lung
O-2	10	MC	Weimaraner	(+)	(−)	(−)	Metastatic Osteosarcoma
O-3	8	MI	Rottweiler	(+)	(+)	(−)	Metastatic Osteosarcoma
O-4	7	MC	Golden Retriever	n/a	(+)	(+)	Metastatic Osteosarcoma
O-5	10	FS	Mixed	n/a	(−)	(+)	Metastatic Osteosarcoma
O-6	5	MC	Labrador Retriever	^a^	^a^	^a^	Metastatic Osteosarcoma
O-9	10	FS	Staffordshire Terrier	(+)	(−)	(+)	Metastatic Osteosarcoma
O-10	8	MC	Great Dane	n/a	(−)	(+)	Metastatic Osteosarcoma
O-11	5	MC	Golden Retriever	(+)	(−)	(+)	Metastatic Osteosarcoma
O-12	10	FS	Mixed	(+)	(+)	(−)	Metastatic Osteosarcoma
O-13	11	FS	Mixed	(+)	(−)	(+)	Metastatic Osteosarcoma
P-1	11	MC	Mixed	(+)	(−)	(−)	Large cell carcinoma
P-3	14	FS	Bichon Frise	(+)	(+)	(+)	Bronchioloalveolar carcinoma
P-4	11	FS	Greyhound	(−)	(+)	(−)	Adenosquamous carcinoma
P-8	9	FS	Scottish Terrier	(+)	(−)	(−)	Bronchioloalveolar carcinoma
P-9	13	MC	Mixed	(+)	(+)	(−)	Bronchioloalveolar carcinoma
P-10	10	MC	Cocker Spaniel	(+)	(−)	(−)	Bronchioloalveolar carcinoma
P-12	10	FS	Boston Terrier	(+)	(−)	(+)	Bronchial gland carcinoma
P-13	10	MC	Boxer	^a^	^a^	^a^	*Acinar Pulmonary adenocarcinoma* ^a^
P-14	12	FS	Gordon Setter	(+)	(+)	(−)	Papillary adenocarcinoma
P-15	14	MC	Mixed	(+)	(−)	(+)	Papillary adenocarcinoma
P-16	14	FS	Welsh Corgi	(+)	(−)	(−)	Large cell carcinoma
P-17	15	MC	Welsh Corgi	(+)	(+)	(+)	Solid adenocarcinoma
P-19	11	FS	Mixed	(+)	(−)	(−)	Bronchioloalveolar carcinoma

*MC* male castrated; *MI* male intact; *FS* female spayed; *FI* female intact; (-) Negative/None/Few/Low; (+) Positive/Mild/Moderate/High/Intense; *n/a* not evaluable/not present

^a^not included in IHC analysis, *italics* = this diagnosis was made by necropsy pathologist, unable to confirm specific subtype, ^b^See materials and methods

Prior to evaluation, all cases (except P-13) were reviewed by DYK to confirm the necropsy report diagnosis. All cases reported as normal lung in the necropsy report were confirmed as such by pathologist review (16 cases). The same was true of all metastatic OSA (10 cases). The NSCLC cases that could be reviewed (12 cases) were further classified as pulmonary adenocarcinoma: papillary type (2 cases) and solid type (1 cases); bronchioloalveolar carcinoma (5 cases); bronchial gland carcinoma (1 case); adenosquamous carcinoma (1 case); and large-cell carcinoma (2 cases) (Table 1).

### COBRA

**Fig. 1:**
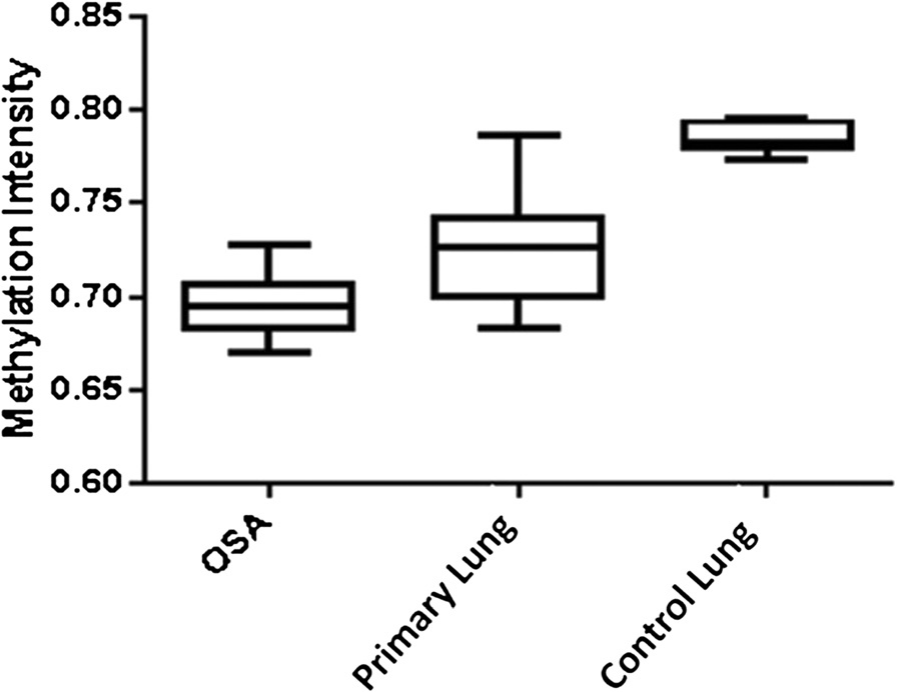
Box Plot of Methylation Intensity of Osteosarcoma, Primary Lung Cancer, and Normal Control Lung. The box incorporates the middle quartiles; the line represents the median value: the whiskers indicate the minimum and maximum values. There is loss of methylation in primary lung cancer (Primary Lung) as compared to normal control lung (Control Lung). There is further loss of methylation in osteosarcoma (OSA). The difference is significant (*p* = 0.0002)

There was a significant difference in methylated band maximum intensities between groups (*p* = 0.0002). Normal lung tissue had the most complete methylation at the evaluated CG site, with primary lung cancer showing relative loss of methylation, and metastatic to lung osteosarcoma being least methylated (Fig. 1).

Bisulfite converted normal canine spleen and Sssi treated and bisulfite converted normal canine spleen were both positive for methylation at this site, suggesting that methylation is expected to be present, even in normal tissues.

### Methylation specific PCR

**Fig. 2:**
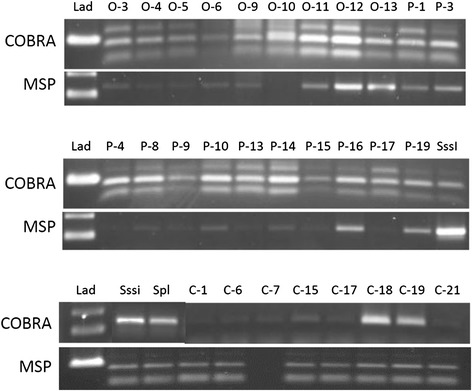
COBRA and MSP for Osteosarcoma, Primary Lung Cancer, and Normal Control Lung. PCR products were run on a 1.5 % agarose gel. Lad = 100 bp ladder. Samples labeled O-X are osteosarcoma, P-X are primary lung cancer, and C-X are control lung. X represents the patient number. Sssi is normal canine spleen DNA methylated in-vitro and Spl is normal canine spleen DNA. Sample C-7 did not amplify by MSP

All but one sample of primary lung cancer (case ID C-7) and all samples of osteosarcoma were amplified by MSP using primers for methylated CG. Normal lung tissue samples also amplified with the methylated MSP primers (Fig. 2).

The same spleen tissue was used to investigate other genes using MSP primers in an unrelated study including *HOXA9*, *DLX13*, *DLX15*, *HOXB5*, and *CDA* and was not methylated in any case (data not shown).

### Western blot

**Fig. 3:**
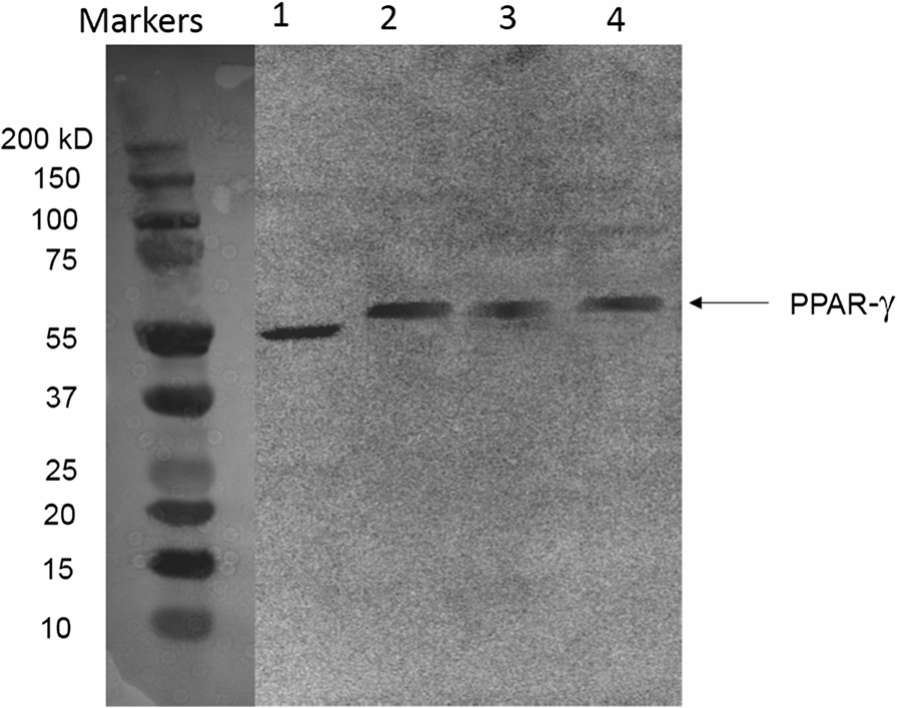
PPARγ Western Blot. Western blot analysis for the expression of PPARγ in fresh tissue samples of human PC3 cells (positive for PPARγ) (1), normal canine placenta (2) and two samples of normal canine lung samples (3 and 4) show protein expression of appropriate weight (57 kDa). The predicted canine protein is 38 amino acids longer than the human protein, which is reflected in the decreased migration of the band on the Western blot (human NM_005037.5 and canine NM_001024632.2). Samples were concurrently formalin fixed and stained with H&E to confirm that they were histologically normal

PPAR-γ protein expression of the appropriate molecular weight of 57 kDa was identified via Western blot in both fresh canine lung and fresh canine placenta (Fig. 3). Human PC3 cells were used as a positive control.

### Immunohistochemistry

**Fig. 4:**
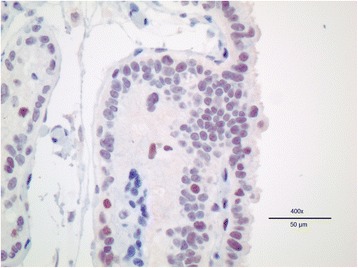
Immunohistochemistry of PPARγ in canine placenta. This 400X view of the canine placenta clearly shows nuclear staining of the trophoblast cells by the antibody

Immunohistochemistry performed on canine placenta (positive control) revealed nuclear staining of trophoblast cells as expected for normal PPAR-γ localization (Fig. 4).

**Fig. 5:**
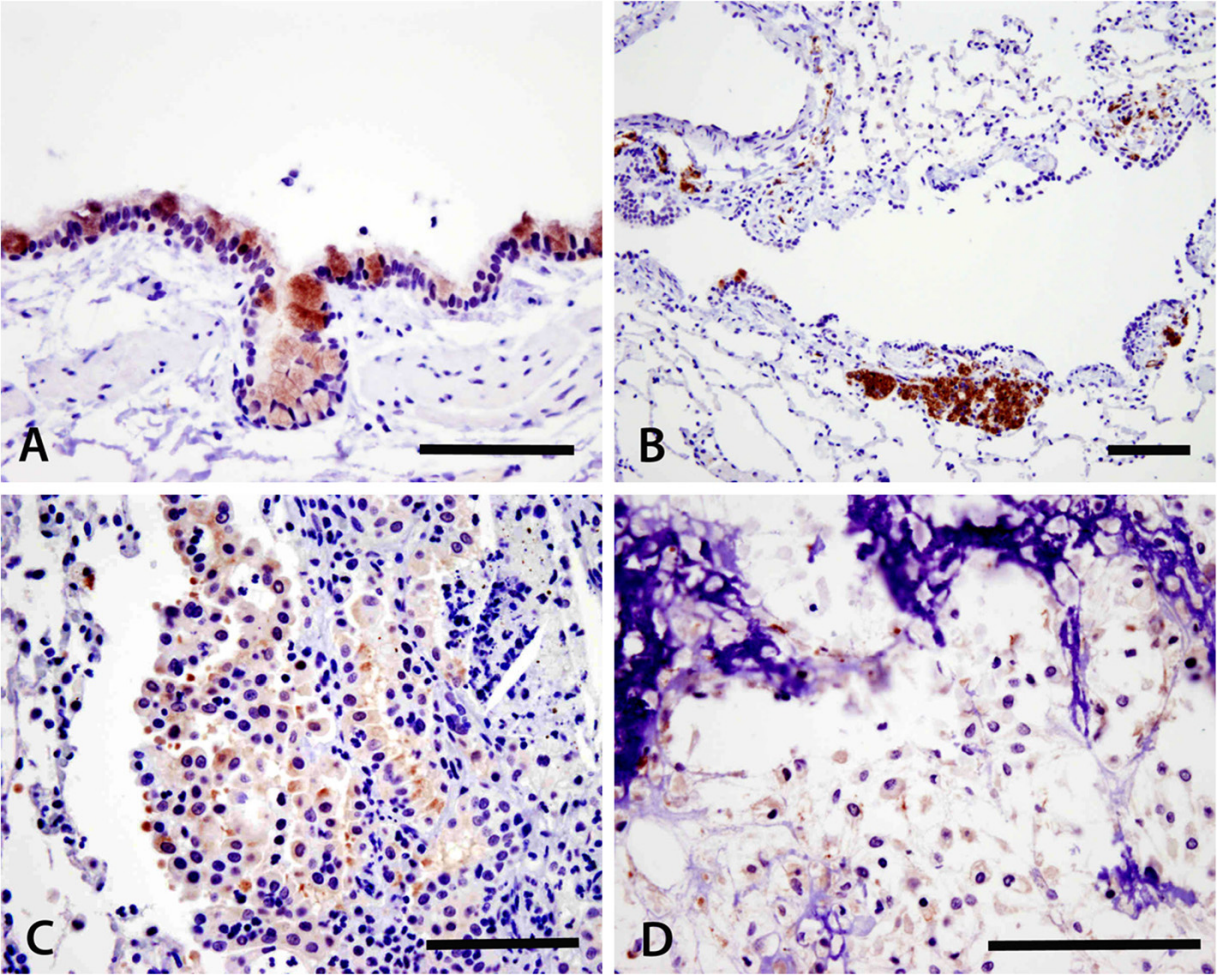
Immunohistochemistry of PPARγ in canine lung tissue. The respiratory epithelium of bronchi and large bronchioles exhibited immunoreactivity of PPARγ, most strongly in the goblet cells (**a**) and engorged peribronchiolar interstitial macrophages that were often filled with carbon particles (**b**). Some of the primary pulmonary adenocarcinomas (**c**) and metastatic osteosarcomas (**d**) expressed positive immunoreactivity. Bar = 100 μm

The majority of bronchi and large bronchioles had strong PPAR-γ positive cytoplasmic immunoreactivity in the epithelium, particularly of goblet cells, and bronchial glands, though not every sample had a large conducting airway present on the slide. In some cases, only tumor tissue was present on the slide, while in others, both tumor and normal tissue were present. Thirty one percent (31 %) of control cases, 66 % of OSA, and 100 % of NSCLC had a large airway available for evaluation, and in every case this airway was positive (Table 1 and Fig. 5a).

Most of the peribronchiolar interstitial macrophages that were markedly swollen with carbon particles were strongly positive. If these macrophages were absent or rare, the cases were scored as 0. The cases containing moderate to high numbers of these macrophages were scored as 1 (Fig. 5b), which occurred in 0 % of control cases, 33 % of OSA, and 42 % of NSCLC. Tumor tissue was immunopositive for PPAR-γ in 66 % of OSA cases and 33 % of NSCLC cases. The tumor tissue was partially stained. The positive stains were observed multifocally within the OSA but often at the periphery in the NSCLC (Table 1 and Fig. 5c-d).

There was no association between methylation of the gene in primary lung cancer cases and expression of the protein in the tumor tissue (*p* = .497), large airways (*p* = 0.931), or macrophages (*p* = 0.931). There was no association between methylation of the gene in metastatic to lung osteosarcoma cases and expression of the protein in the tumor tissue (*p* = .429), large airways (*p* = 1.0), or macrophages (*p* = 0.571).

## Discussion

**Fig. 6:**
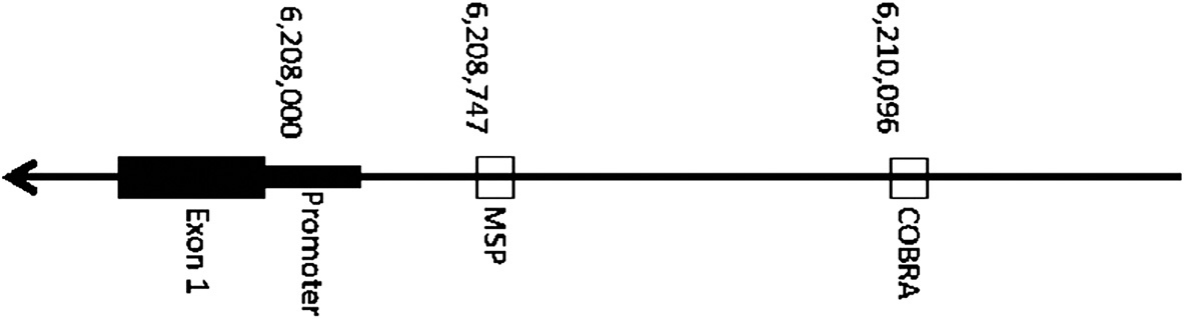
Schematic representation PPARγ gene. The location of the promoter, as well as the sites of interrogation by MSP and COBRA primers is represented

In general, it is believed that in carcinogenesis there is a shift in the epigenetic profile of cells in which genome wide hypomethylation develops, accompanied by regional hypermethylation, specifically in promoter regions of genes. When hypermethylation occurs in promoter regions of tumor suppressor genes, transcription machinery often is impaired, and therefore gene transcription is inhibited. As *PPAR-γ* is believed to act as a tumor suppressor in lung cancer development, we hypothesized that *PPAR-γ* promoter would be hypermethylated in all tumor samples, and that frequency of hypermethylation would be greater in the more biologically aggressive cancer (metastatic OSA) than in primary lung tumors. Our findings are the opposite, in that methylation of PPAR-γ was reduced in primary lung cancer as compared to normal lung, and even further loss of methylation occurred in aggressive metastatic to lung osteosarcoma. The interrogated region was selected based on prior data generated in our laboratory identifying methylation at this locus in canine lymphoma. The methylation previously was identified in spite of a lack of a formal CpG island in the region. It does not appear that the interrogated CG dinucleotides are active in gene expression in the evaluated tissues (Fig. 6). These CG dinucleotides may then act as genomic CGs, as opposed to promoter CGs, and therefore have little impact on gene expression. If acting as genomic CGs, loss of methylation with increasing malignancy would be expected, as was identified here. It is also possible that the queried CGs were too far from the transcription start site to modify gene expression [24]. It is also possible, but less likely, that PPAR-γ is serving as a tumor promoter, instead of a tumor suppressor, and that methylation decreases with increasing malignancy, although these data did not demonstrate such a relationship. It is unclear as to why C-7 would not amplify by MSP, though this was the case with multiple attempts at the experiment. In spite of measurable DNA in the sample, it is most likely that the sample was partially degraded, making it difficult to amplify with MSP primers, or that the amplification is below the visible limit of detection.

Bisulfite treatment of DNA results in conversion of cytosine to uracil, but leaves 5-methylcytosine unaltered. Sssi treatment converts all cytosines to 5-methylcytosine.

In these samples, both bisulfite converted normal canine spleen and Sssi treated and bisulfite converted normal canine spleen were positive for methylation. The most logical explanation for this finding is native hypermethylation at the investigated locus. The methylation status of *PPAR-γ* in normal canine spleen has not been reported previously, and to the authors’ knowledge has not been investigated in normal human spleen. PPAR-γ protein expression is reported to be high in the rat spleen however [3, 25]. In unpublished data evaluating the methylation status of many other tumor suppressor genes, DNA from spleen tissue of the same dog did not show hypermethylation. The repeatable nature of the finding at this locus, along with negative tumor tissues, supports its validity.

The immunohistochemistry results of this study are the first to describe PPAR-γ protein localization in normal canine lung and canine lung cancer. Studies of PPAR-γ expression in other species are similar to the findings here. Cytoplasmic PPAR-γ expression was found in the epithelium of large conducting airways [26] and in alveolar macrophages [27, 28]. PPAR-γ expression has also been described in alveolar epithelial cells in some studies, but was not identified here [27, 28]. Reasons for differences across species could be due to true differences in PPAR-γ expression within the lung, due to differences in tissue processing and therefore antibody binding, or due to differences in sensitivity of detection.

This is the first study to describe immunohistochemical positivity for either metastatic or primary lung tumors in the dog. The incidence of IHC positive cases of NSCLC in dogs (33 %) is similar to studies in human NSCLC, which found that 42–45 % of cases were positive [29, 30]. As in human NSCLC [29, 30], the cytoplasmic distribution was also found here. It is unclear why PPAR-γ is primarily cytoplasmic in both human and canine NSCLC, however various other carcinomas have described primarily cytoplasmic PPAR-γ staining [31, 32]. One proposed mechanism by which PPAR-γ is trapped in the cytoplasm is nitration, which inhibits translocation into the nucleus. This has been demonstrated in a macrophage-like cell line, although further investigation would be necessary to determine if this is the mechanism at play here [33]. In some human NSCLC studies, it has been found that expression of PPAR-γ correlated with tumor type and grade [29], while in other studies no such correlation existed [30]. Due to the small number of samples in this study, no attempt was made to correlate histologic subtype or tumor grade to PPAR-γ positivity. This would be a next reasonable step for future studies where larger patient numbers could be obtained.

To the authors’ knowledge, IHC evaluation of PPAR-γ in primary or metastatic OSA has not been described in any species. Studies of human OSA cell lines have shown increased PPAR-γ mRNA message, suggesting that this tumor does express PPAR-γ [34]. It is also known that PPAR-γ is important for osteoblast differentiation [35, 36], and there is some suggestion that PPAR-γ agonists could inhibit OSA proliferation and induce apoptosis [37]. From the present findings, it is impossible to speculate on the role that PPAR-γ may play in OSA tumorigenesis, but it does provide the first investigation of PPAR-γ expression in metastatic OSA in the dog. In addition, it would be interesting to compare the primary tumor from OSA samples to the corresponding metastatic pulmonary lesions, however this was not possible with the cases available, as the primary tumors were often removed prior to necropsy, making tissues unavailable for comparison.

There are some limitations to the materials in the present study. An attempt was made during case selection to age-match controls to the tumor groups through exclusion of cases < 5 years of age. This arbitrary age was selected to remove very young dogs from the control group, as methylation of tissues has been shown to increase with age [38]. This method allowed for age-matching between the OSA group and the control group, however the NSCLC group was significantly older. These age differences are, however, consistent with age at diagnosis in other reports, in which the average age at diagnosis of primary lung tumors is 11 years whereas the average age at OSA diagnosis is 7–9 years [39, 40]. It is very uncommon for animals in the patient database at or around 11 years of age to be submitted for elective necropsy and have histologically normal lung tissue on necropsy and no other tumor capable of metastasis. This explains why an older group of control animals was not identified. Additionally, the tumor type distribution for primary lung tumors is also consistent with what has previously been reported, with adenocarcinoma and bronchoalveolar carcinoma being the most common primary lung tumors of dogs [41, 42].

There was some sample loss in comparing cases that had adequate tissue for IHC as compared to cases that had adequate tissue for COBRA and MSP. The reason for the loss of available tissue for epigenetic studies is a direct reflection of the type/size of samples used for this study. All tissues in this study were obtained from archived patient tissues. All samples were obtained from a veterinary diagnostic laboratory, and as such were considered a part of the patient medical record, therefore, the majority of the tissue had to be preserved to maintain the integrity of the medical record. For all IHC samples, only a single 5 um slice needed to be harvested from the archived block, and did not result in deterioration of the patient sample, so could be performed for most cases identified. For DNA harvest, a maximum of 40um slice of tissue could be trimmed from the tissue before interfering with preservation of the patient medical record. In cases of normal control lung, often only small pieces of lung tissue were included in the paraffin embedded block, and were also often combined with up to five other tissues from the same patient. In these cases, only lung tissue was processed. If adequate DNA could not be harvested after a single extraction, the blocks could not be disrupted further. For tumor cases, often the entire paraffin embedded block consisted of the pathologic sample, so there was a high percentage of cases that both IHC and DNA extraction could be performed due to the large amount of preserved tissue. This is the same reason that not all tissue had a large airway available for evaluation of PPAR-γ expression via immunohistochemistry. In general, with the smaller samples available for normal control lung, it is not surprising that many sections did not have large airways present for evaluation. In future studies, using tissue collected specifically for the designed study could result in a higher percentage of cases that have adequate DNA available for methylation analysis as well as ensure that all types of lung tissue (conducting airways and alveoli) would be available for evaluation by immunohistochemistry.

The most important finding of this study is the demonstration of lung tumor positivity for PPAR-γ in dogs with NSCLC and metastatic OSA. There are currently no published successful medical treatments for either of these conditions in the dog. Dogs with high tumor stage or lymph node metastasis with primary lung tumors have median survival times of < 2 months [43]. Dogs with metastatic OSA have a median survival of < 2-3 months, even with treatment [44]. PPAR-γ agonists including rosiglitazone and pioglitazone have been studied in the dog, and therapeutic doses and dose-limiting toxicities are published [45, 46]. In addition, a phase I clinical trial of oral rosiglitazone and carboplatin in cancer-bearing dogs showed that this combination was safe [23]. The findings of this study provide rationale to suggest that dogs with primary lung tumor and metastatic osteosarcoma could serve as a population which would be appropriate to treat with PPAR-γ agonists, and that expression of PPAR-γ can be demonstrated in these tumor types.

## Conclusions

The results of this study show that PPAR-γ protein is expressed in normal canine lung tissue and in canine primary and metastatic to lung cancer. This report is the first to demonstrate that the frequency of PPAR-γ protein expression in canine primary lung cancer is similar to the frequency described in human NSCLC. These data supports the use of PPAR-γ agonists in this subset of veterinary patients, and suggest that the dog may serve as an appropriate translational model for the study of PPAR-γ agonist use in lung cancer treatment. There are differences in PPAR-γ methylation among normal lung, primary lung cancer, and metastatic osteosarcoma in the dog, but these differences do not relate to protein expression levels.

## Methods

### Tissue procurement

The University of Missouri Veterinary Diagnostic Laboratory (VMDL) UVIS database was queried from 2005 to 2011 to identify tissue samples for analysis. Because no tissue was collected from a live animal or human for purposes of the study, and all were de-identified of client and patient information beyond age, sex, and breed, no ethical approvals were required for the study. All necropsy submissions to the VMDL include permission for use of collected tissues in future research.

For selection of tumor-bearing cases, the database was searched for dogs with a diagnosis of OSA, metastatic OSA, pulmonary carcinoma, pulmonary adenocarcinoma or bronchiolar carcinoma. Cases were included if a sample or biopsy of the affected lung tissue was obtained and submitted for diagnosis and archiving. The hematoxylin and eosin-stained (H&E) slides from these cases were reviewed by a single pathologist (DYK) to confirm that lung tissue was present and support the original diagnosis of either metastatic OSA or NSCLC. DNA was extracted from paraffin-embedded lung tissue blocks using a commercially available DNA extraction kit (NucleoSpin Tissue, Machery-Nagel. Düren, Germany).

For normal lung control animals, the database was searched for any animals for which a necropsy was performed and non-diseased lung tissue was reported in the necropsy report and for which formalin fixed and embedded paraffin blocks were archived. Cases were excluded if a metastatic or primary lung tumor was identified, if the dog had cancer elsewhere that could reasonably be expected to metastasize, or if significant lung disease was reported. Cases were also excluded if significant liver or kidney disease was noted in the medical record to prevent detection of changes in the methylation status of DNA due to systemic disease. Significant liver and kidney disease was defined as a diagnosis code entry in the patient record to include inflammatory/infectious conditions (any hepatitis or nephritis), any neoplasia in these organs, or any diagnosis code of significant organ dysfunction or failure (liver failure, hepatopathy, renal failure, nephropathy). In addition, if these diagnoses were noted in the necropsy report, but not entered as a diagnostic code, the cases were also excluded. Finally, cases with an age < 5 years were excluded in an attempt to age match the control cases, and exclude methylation changes with age as a confounder. The slides and tissues were then collected, reviewed, and processed as described above.

For both immunohistochemistry and methylation analysis, when more than one tissue type was present in the archived tissue block, only the lung tissue was collected for analysis by trimming away all non-lung derived tissue. Thirteen cases of metastatic to lung OSA (cases O-1 thru 13), 19 cases of NSCLC (cases P-1 thru 19), and 22 control cases were identified (cases C-1 thru 22). From these cases identified for inclusion in the study, 10 cases of OSA, 12 cases of NSCLC, and 16 cases of normal lung had tissue available for pathologist review of the H&E stained slides (Table 1 column 8). From the identified cases, 9 cases of OSA (case 0–6 unavailable), 12 cases of NSCLC, and 16 control lung had adequate samples available for immunohistochemistry. Also from the identified cases, 9 cases of OSA (case O-2 unavailable), 12 cases of NSCLC, and 8 cases (C-1, 6, 7, 15, 17, 18, 19, 21) of control lungs had enough DNA to perform COBRA/MSP.

### DNA extraction and methylation analysis

DNA was harvested using NucleoSpin Tissue (Machery-Nagel. Düren, Germany) according to the provided protocols. Briefly, xylene and ethanol was used to remove paraffin-wax from the embedded tissue samples. The cells were lysed and proteins digested with Proteinase K, and ethanol was added to adjust DNA binding conditions. The solution was then centrifuged in a spin column provided with the kit to bind the total DNA. The DNA was washed twice and finally eluted as a highly pure product.

COBRA: The MethPrimer (http://www.urogene.org/cgi-bin/methprimer/methprimer.cgi) website was used to identify an appropriate primer using as input the canine gene located at the 5′ end of the PPAR-γ (RefSeq NM_001024632.2) chr20:6,210,096-6,210,218 CanFam2 [47]. The product size of these primers is 124 bp and contains one TaqaI cut site yielding fragments of 86 and 38 bp. The primers used for COBRA were (5′ to 3′): forward, TTTTTAGAAGTGTTTGAATTATTGGG and reverse, AAAAACAAACTCCATACAAAAAACC. PCR was performed at an annealing temperature of 60 °C for 60 s, an extension temperature of 72 °C for 60 s, and a melting temperature of 95 °C for 60 s, repeating for 36 cycles. The PCR product was purified with the NucleoSpin Gel and PCR Clean-up kit (Macherey-Nagel, Bethlehem, PA) and eluted in 20 μL of HyPure water. For TaqaI digestion, 10 μL of PCR product was added to 2.5 μL of Buffer 2, 1 μL of TaqaI, and 11.5 μL of HyPure water, and incubated at 60 °C for 4 h. A schematic representation of the PPAR-γ gene is shown in Fig. 6.

MSP was performed on the lung tissue samples described above using PPAR-γ primers created in the region 5′ from the promoter from MethPrimer (http://www.urogene.org/cgi-bin/methprimer/methprimer.cgi). Primers were against bisulfite-treated DNA located at chr20:6,206,804-6,210,823. This region was identified in other, unrelated experiments to be hypermethylated in canine cancers (data not shown). The forward and reverse sequences were AATTGATTTATATTGATAGGTTGGC and TTCCATACTAAAATTTAACACGAC respectively. Using bisulfite treated canine DNA from normal spleen, the conditions for MSP were optimized. The methylated primer was used to amplify the region with an annealing temperature appropriate to the primer design for 30 s, an extension temperature of 72 °C for 30 s, and a melting temperature of 95 °C for 15 s, repeating for 32 cycles. The PCR products were run on a 1.5 % agarose gel with Gel Red. The controls for COBRA and MSP consisted of bisulfite converted DNA from normal canine spleen, and DNA from a normal canine spleen methylated in vitro using SssI, then bisulfite converted (to induce methylation of the sample).

Methylation intensity of the COBRA assay was determined using Image J. Regions of interest were created around the region of the primary band (123 bp) and each cut band (85 bp and 39 bp). A ratio was calculated with the added intensities of the cut bands (methylated) over the intensity of the primary band (unmethylated). The higher the ratio, the greater the relative proportion of methylation in the sample.

### Immunohistochemistry

From the archived tissues of adequate condition, 9 cases of OSA, 12 cases of NSCLC, and 16 control lung samples were processed for PPAR-γ immunohistochemistry evaluation and scoring. For immunohistochemistry, the paraffin blocks of the selected cases were sectioned by 5 μm. Anti-human PPAR-gamma rabbit polyclonal antibody (sc-7196, Santa Cruz Biotechnology, Dallas, TX) served as the primary antibody. Heat-induced antigen retrieval using citrate buffer (0.01 M, pH 6.0) and EnVision™+ system (Dako, Carpinteria, CA) were used. The immunoreactivity was visualized by using Romulin AEC Chromogen (Biocare Medical, Concord, CA) and haematoxylin was used as counterstain. In each case, negative controls were included in which the primary antibody was excluded. For a positive control, fresh canine placenta was used, as this has been previously demonstrated as positive in the dog [48].

### Western blot

Western blot analysis was performed on fresh canine lung and fresh canine placenta confirmed by H&E as microscopically non-diseased tissue as well as human PC3 cells. The fresh canine lung was obtained from a dog that had been euthanized for unrelated causes and submitted to VMDL for necropsy. The fresh canine placenta was obtained from a local breeder and client of the University of Missouri VMTH who volunteered the tissues immediately after whelping. The human PC3 cells were obtained from the American Type Culture Collection (www.atcc.org). No special permissions or ethical approval was required for use of the fresh canine and human tissue. Tissue lysate was treated with M-PER mammalian protein extraction reagent (Thermo-Fisher, Rockford, IL) as per manufacturer’s instruction. Protein concentration was determined using the Bradford method (Thermo-Fisher,Rockford, IL). Equal amounts of protein (~ 40 μg) were separated on a 10 % SDS polyacrylamide gel and transferred to a nitrocellulose membrane (Bio-Rad, Hercules, CA). The blots were blocked at room temperature for 1 h using Tris-saline buffer (TBS) containing 0.1 % Tween 20 and 10 % nonfat milk. The membrane was further incubated with primary antibody for PPAR-γ (sc-7196, Santa Cruz Biotechnology) overnight at 4 °C. After washing with TBS, the membrane was incubated with a horseradish peroxidase-labeled secondary antibody and visualized with a chemiluminescence detection kit (Thermo-Fisher, Rockford, IL). The blot was imaged using a Kodak imaging station (Carestream Health, Rochester, NY).

### Evaluation of immunohistochemistry

The slides were reviewed and scored by a pathologist (DYK). Some epithelial cells and goblet cells of bronchi and large bronchioles, some bronchial glands, peribronchiolar interstitial macrophages that often were filled with carbon particles, and tumor cells were positive for PPAR-γ IHC expression. Each structure/cell type above was scored separately. The absence of positive staining in bronchi/bronchioles was scored 0 (−) and presence was scored 1 (+). For the peribronchiolar interstitial macrophages, 0 (−) represented zero to few positive macrophages and 1 (+) represented moderate to high numbers of immunopositive macrophages. For tumor cells 0 (−) represented no positivity and 1 (+) represented mild to intense immunopositivity.

### Statistical analysis

Age and gender comparisons were made using an ANOVA on Ranks. Methylation intensities were compared in categorical variables using a Mann-Whitney *U* test. Association between gene methylation and tissue expression were made using an ANOVA on Ranks. *P*-values less than or equal to 0.05 were considered significant.

## Availability of supporting data

All necessary data is included in the body of this manuscript.

## Abbreviations

COBRA: Combined bisulfite restriction analysis
IHC: Immunohistochemistry
MSP: Methylation specific PCR
NSCLC: Non-small cell lung cancer
OSA: Osteosarcoma
PPARγ: Peroxisome proliferator activated receptor
SCLC: Small cell lung cancer

## References

[1] Kliewer SA, Sundseth SS, Jones SA, Brown PJ, Wisely GB, Koble CS, Devchand P, Wahli W, Willson TM, Lenhard JM, Lehmann JM (1997). Fatty acids and eicosanoids regulate gene expression through direct interactions with peroxisome proliferator-activated receptors α and γ. Proc Natl Acad Sci.

[2] Chinetti G, Fruchart J-C, Staels B (2000). Peroxisome proliferator-activated receptors (PPARs): Nuclear receptors at the crossroads between lipid metabolism and inflammation. Inflamm Res.

[3] Ricote M, Li AC, Willson TM, Kelly CJ, Glass CK (1998). The peroxisome proliferator-activated receptor-γ is a negative regulator of macrophage activation. Nature.

[4] Grommes C, Landreth GE, Heneka MT (2004). Antineoplastic effects of peroxisome proliferator-activated receptor gamma agonists. Lancet Oncol.

[5] Peters JM, Shah YM, Gonzalez FJ (2012). The role of peroxisome proliferator-activated receptors in carcinogenesis and chemoprevention. Nat Rev Cancer.

[6] Han SW, Roman J (2008). Activated PPARgamma targets surface and intracellular signals that inhibit the proliferation of lung carcinoma cells. PPAR Res.

[7] Keshamouni VG, Reddy RC, Arenberg DA, Joel B, Thannickal VJ, Kalemkerian GP, Standiford TJ (2004). Peroxisome proliferator-activated receptor-γ activation inhibits tumor progression in non-small-cell lung cancer. Oncogene.

[8] Li H, Weiser-Evans MCM, Nemenoff R. Anti- and protumorigenic effects of PPAR*γ;* in lung cancer progression: a double-edged sword. PPAR Res. 2012;2012. 10.1155/2012/362085 PMC3425863 22934105

[9] Jemal A, Siegel R, Xu J, Ward E (2010). Cancer statistics, 2010. CA Cancer J Clin.

[10] Chang T-H, Szabo E (2000). Induction of differentiation and apoptosis by ligands of peroxisome proliferator-activated receptor γ in non-small cell lung cancer. Cancer Res.

[11] Tsubouchi Y, Sano H, Kawahito Y, Mukai S, Yamada R, Kohno M, Inoue K, Hla T, Kondo M (2000). Inhibition of human lung cancer cell growth by the peroxisome proliferator-activated receptor-γ agonists through induction of apoptosis. Biochem Biophys Res Commun.

[12] Rowell JL, McCarthy DO, Alvarez CE (2011). Dog models of naturally occurring cancer. Trends Mol Med.

[13] Breen M, Modiano JF (2008). Evolutionarily conserved cytogenetic changes in hematological malignancies of dogs and humans – man and his best friend share more than companionship. Chromosome Res.

[14] Paoloni M, Khanna C (2008). Translation of new cancer treatments from pet dogs to humans. Nat Rev Cancer.

[15] Kraegel SA, Gumerlock PH, Dungworth DL, Oreffo VIC, Madewell BR (1992). K-ras activation in Non-small cell lung cancer in the dog. Cancer Res.

[16] Hifumi T, Miyoshi N, Kawaguchi H, Nomura K, Yasuda N (2010). Immunohistochemical detection of proteins associated with multidrug resistance to anti-cancer drugs in canine and feline primary pulmonary carcinoma. J Vet Med Sci Jpn Soc Vet Sci.

[17] Khanna C, Vail DM (2003). Targeting the lung: preclinical and comparative evaluation of anticancer aerosols in dogs with naturally occurring cancers. Curr Cancer Drug Targets.

[18] Hershey IDK AE (1999). Inhalation chemotherapy for macroscopic primary or metastatic lung tumors: proof of principle using dogs with spontaneously occurring tumors as a model. Clin Cancer Res Off J Am Assoc Cancer Res.

[19] Sozmen M, Kabak YB, Gulbahar MY, Gacar A, Karayigit MO, Guvenc T, Yarim M (2013). Immunohistochemical characterization of peroxisome proliferator-activated receptors in canine normal testis and testicular tumours. J Comp Pathol.

[20] Paciello O, Borzacchiello G, Varricchio E, Papparella S (2007). Expression of peroxisome proliferator-activated receptor gamma (PPAR-γ) in canine nasal carcinomas. J Vet Med Ser A.

[21] Toseland CDN, Campbell S, Francis I, Bugelski PJ, Mehdi N (2001). Comparison of adipose tissue changes following administration of rosiglitazone in the dog and rat. Diabetes Obes Metab.

[22] Frazier SA, McKemie DS, Guerrero TA, Skorupski KA, Rodriguez CO (2011). Evaluation of an extractionless high-performance liquid chromatography-tandem mass spectrometry method for detection and quantitation of rosiglitazone in canine plasma. Am J Vet Res.

[23] Allstadt Frazier S, McKemie DS, Guerrero TA, LaChapelle H, Skorupski KA, Kass PH, Rodriguez CO (2014). Phase I clinical trial of oral rosiglitazone in combination with intravenous carboplatin in cancer-bearing dogs. Vet Comp Oncol.

[24] Jones PA (2012). Functions of DNA methylation: islands, start sites, gene bodies and beyond. Nat Rev Genet.

[25] Braissant O, Foufelle F, Scotto C, Dauça M, Wahli W (1996). Differential expression of peroxisome proliferator-activated receptors (PPARs): tissue distribution of PPAR-alpha, -beta, and -gamma in the adult rat. Endocrinology.

[26] Simon DM, Arikan MC, Srisuma S, Bhattacharya S, Tsai LW, Ingenito EP, Gonzalez F, Shapiro SD, Mariani TJ (2006). Epithelial cell PPARγ contributes to normal lung maturation. FASEB J.

[27] Liu D, Xiong Zeng B, Shang Y (2006). Decreased expression of peroxisome proliferator-activated receptor γ in endotoxin-induced acute lung injury. Physiol Res.

[28] Ameshima S, Golpon H, Cool CD, Chan D, Vandivier RW, Gardai SJ, Wick M, Nemenoff RA, Geraci MW, Voelkel NF (2003). Peroxisome proliferator-activated receptor gamma (PPARγ) expression is decreased in pulmonary hypertension and affects endothelial cell growth. Circ Res.

[29] Theocharis S, Kanelli H, Politi E, Margeli A, Karkandaris C, Philippides T, Koutselinis A (2002). Expression of peroxisome proliferator activated receptor-gamma in non-small cell lung carcinoma: correlation with histological type and grade. Lung Cancer.

[30] Giaginis C, Politi E, Alexandrou P, Sfiniadakis J, Kouraklis G, Theocharis S (2012). Expression of peroxisome proliferator activated receptor-gamma (PPAR-γ) in human non-small cell lung carcinoma: correlation with clinicopathological parameters, proliferation and apoptosis related molecules and patients’ survival. Pathol Oncol Res.

[31] Mukunyadzi P, Ai L, Portilla D, Barnes EL, Fan C-Y (2003). Expression of peroxisome proliferator-activated receptor gamma in salivary duct carcinoma: immunohistochemical analysis of 15 cases. Mod Pathol.

[32] Zhang GY, Ahmed N, Riley C, Oliva K, Barker G, Quinn MA, Rice GE (2004). Enhanced expression of peroxisome proliferator-activated receptor gamma in epithelial ovarian carcinoma. Br J Cancer.

[33] Shibuya A, Wada K, Nakajima A, Saeki M, Katayama K, Mayumi T, Kadowaki T, Niwa H, Kamisaki Y (2002). Nitration of PPARgamma inhibits ligand-dependent translocation into the nucleus in a macrophage-like cell line, RAW 264. FEBS Lett.

[34] Haydon RC, Zhou L, Feng T, Breyer B, Cheng H, Jiang W, Ishikawa A, Peabody T, Montag A, Simon MA, He T-C (2002). Nuclear receptor agonists as potential differentiation therapy agents for human osteosarcoma. Clin Cancer Res.

[35] Haydon RC, Luu HH, He T-C (2007). Osteosarcoma and osteoblastic differentiation: a new perspective on oncogenesis. Clin Orthop.

[36] Tang N, Song W-X, Luo J, Haydon RC, He T-C (2008). Osteosarcoma development and stem cell differentiation. Clin Orthop.

[37] Wagner ER, He B-C, Chen L, Zuo G-W, Zhang W, Shi Q, et al. Therapeutic Implications of PPARγ in Human Osteosarcoma. PPAR Res 2010, 2010. doi: 10.1155/2010/956427 10.1155/2010/956427 PMC2825651 20182546

[38] Fuke C, Shimabukuro M, Petronis A, Sugimoto J, Oda T, Miura K, Miyazaki T, Ogura C, Okazaki Y, Jinno Y (2004). Age related changes in 5-methylcytosine content in human peripheral leukocytes and placentas: an HPLC-based study. Ann Hum Genet.

[39] Ru G, Terracini B, Glickman LT (1998). Host related risk factors for canine osteosarcoma. Vet J.

[40] Withrow SJ, Vail DM, Page RL (2013). Withrow and MacEwen’s small animal clinical oncology.

[41] Griffey SM, Kraegel SA, Madewell BR (1998). Rapid detection of K-ras gene mutations in canine lung cancer using single-strand conformational polymorphism analysis. Carcinogenesis.

[42] Ogilvie GK, Haschek WM, Withrow SJ, Richardson RC, Harvey HJ, Henderson RA, Fowler JD, Norris AM, Tomlinson J, McCaw D (1989). Classification of primary lung tumors in dogs: 210 cases (1975–1985). J Am Vet Med Assoc.

[43] Polton GA, Brearley MJ, Powell SM, Burton CA (2008). Impact of primary tumour stage on survival in dogs with solitary lung tumours. J Small Anim Pract.

[44] Boston SE, Ehrhart NP, Dernell WS, Lafferty M, Withrow SJ (2006). Evaluation of survival time in dogs with stage III osteosarcoma that undergo treatment: 90 cases (1985–2004). J Am Vet Med Assoc.

[45] Boileau C, Martel-Pelletier J, Fahmi H, Mineau F, Boily M, Pelletier J-P (2007). The peroxisome proliferator–activated receptor γ agonist pioglitazone reduces the development of cartilage lesions in an experimental dog model of osteoarthritis: In vivo protective effects mediated through the inhibition of key signaling and catabolic pathways. Arthritis Rheum.

[46] Nemoto S, Razeghi P, Ishiyama M, Freitas GD, Taegtmeyer H, Carabello BA (2005). PPAR-γ agonist rosiglitazone ameliorates ventricular dysfunction in experimental chronic mitral regurgitation. Am J Physiol - Heart Circ Physiol.

[47] Li L-C, Dahiya R (2002). MethPrimer: designing primers for methylation PCRs. Bioinforma Oxf Engl.

[48] Kowalewski MP, Meyer A, Hoffmann B, Aslan S, Boos A (2011). Expression and functional implications of Peroxisome Proliferator—Activated Receptor Gamma (PPARγ) in canine reproductive tissues during normal pregnancy and parturition and at antiprogestin induced abortion. Theriogenology.

